# Correction: Genetic diversity analysis of the invasive gall pest Leptocybe invasa (Hymenoptera: Apodemidae) from China

**DOI:** 10.1371/journal.pone.0316116

**Published:** 2024-12-16

**Authors:** Xin Peng, Hantang Wang, Chunhui Guo, Ping Hu, Lei Xu, Jing Zhou, Zhirou Ding, Zhende Yang

The family name of species ""Hymenoptera: Apodemidae"" in the article title should have been ""Hymenoptera: Eulophidae"". The correct title is: Genetic diversity analysis of the invasive gall pest (italic)Leptocybe invasa (Hymenoptera: Eulophidae) from China. The correct citation is: Peng X, Wang H, Guo C, Hu P, Xu L, Zhou J, et al. (2021) Genetic diversity analysis of the invasive gall pest Leptocybe invasa (Hymenoptera: Eulophidae) from China. PLoS ONE 16(10): e0258610. https://doi.org/10.1371/journal.pone.0258610

In the Introduction section, there is an error in the first sentence of the second paragraph. The correct sentence is: Leptocybe invasa Fisher et LaSalle (Hymenoptera: Eulophidae) is an invasive gall pest that damages more than 30 species of Eucalyptus and mainly harms the branches and leaves of eucalyptus, causing typical lumplike galls on the veins, petioles and shoots of new leaves.

In the Introduction section, there is an error in the sixth sentence of the second paragraph. The correct sentence is: Currently, it has spread to Guangxi, Hainan, Guangdong, Fujian, Sichuan, Jiangxi, Yunnan, Taiwan.

In the Introduction section, there is an error in the third sentence of the third paragraph. The correct sentence is: Lineages A and B are distributed together in Laos, Thailand, Vietnam, South Africa.

In the Introduction section, there is an error in the fourth sentence of the fourth paragraph. The correct sentence is: In recent years, L. invasa males have been reported in Turkey (male/female ratio: 1:124) [13], India [14], China (male/female ratio: 1:126& 1:195) [15], Taiwan [16], Thailand [17] and other places successively.
