# Supplementary material for: Genetic diversity analysis of the invasive gall pest Leptocybe invasa (Hymenoptera: Apodemidae) from China
Source: PLoS One. 2021 Oct 14;16(10):e0258610. doi: 10.1371/journal.pone.0258610 (PMC8516283; doi:10.1371/journal.pone.0258610)
Supplement: S1 Fig — (DOCX) [file pone.0258610.s001.docx]

**S1 Fig. *L. invasa* SSR capillary electrophoresis peak**

Primer number: c120771 Alleles size: 248、252 Fluorescent label: FAM

| 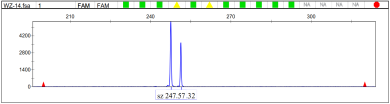 | 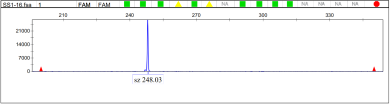 | 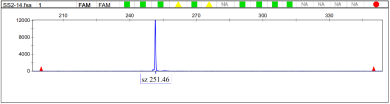 |
| --- | --- | --- |

Primer number: c124062 Alleles size: 145、155 Fluorescent label: FAM

| 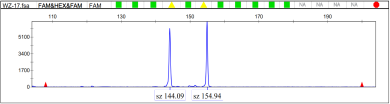 | 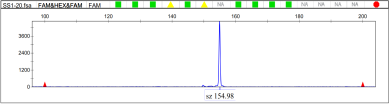 | 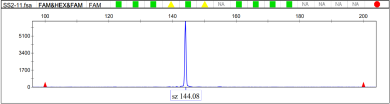 |
| --- | --- | --- |

Primer number: c120888 Alleles size: 233、239 Fluorescent label: HEX

| 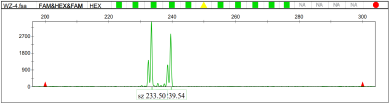 | 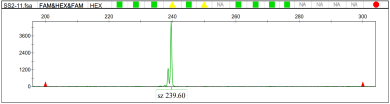 | 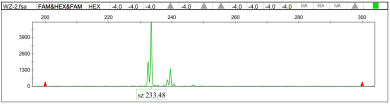 |
| --- | --- | --- |

Primer number: c121460 Alleles size: 196、199、217 Fluorescent label: ROX

| 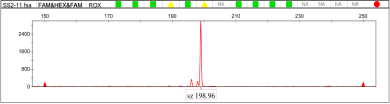 | 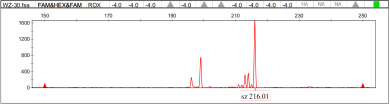 | 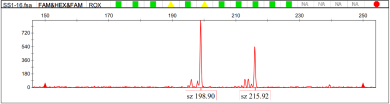 |
| --- | --- | --- |
| 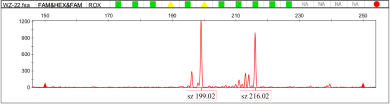 | 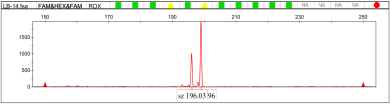 |  |

Primer number: c69914 Alleles size: 198、200、202、204 Fluorescent label: FAM

| 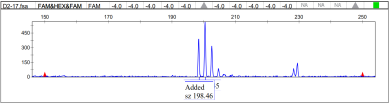 | 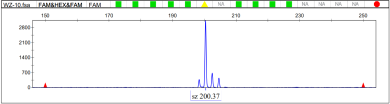 | 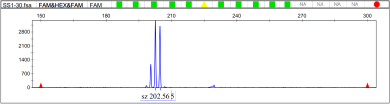 |
| --- | --- | --- |
| 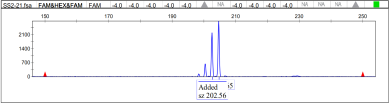 |  |  |

Primer number: c121749 Alleles size: 249、258 Fluorescent label: ROX

| 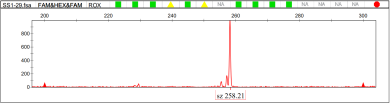 | 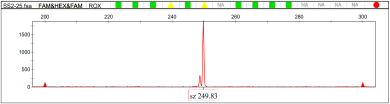 | 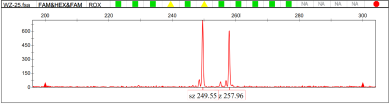 |
| --- | --- | --- |

Primer number: c127471 Alleles size: 220、229 Fluorescent label: TAMRA

| 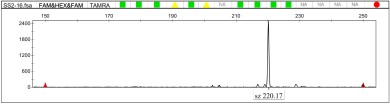 | 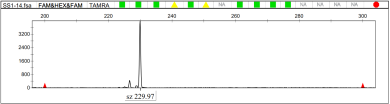 | 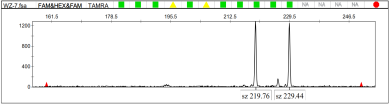 |
| --- | --- | --- |

Primer number: c123946 Alleles size: 237、240、246、249 Fluorescent label: HEX

| 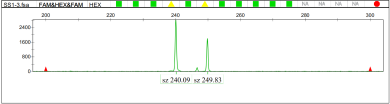 | 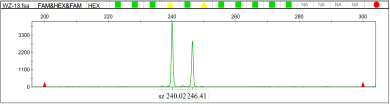 | 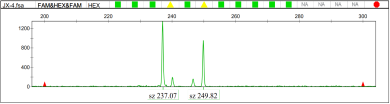 |
| --- | --- | --- |
| 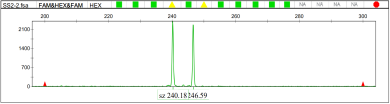 |  |  |

Primer number: LiSS5 Alleles size: 351、354 Fluorescent label: ROX

| 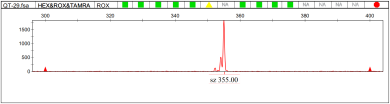 | 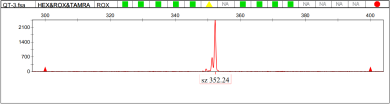 | 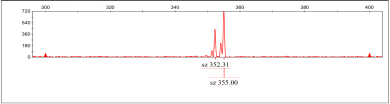 |
| --- | --- | --- |

Primer number: LiSS13 Alleles size: 141、149 Fluorescent label: FAM

| 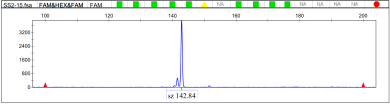 | 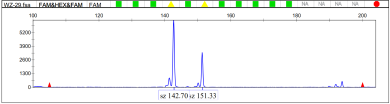 | 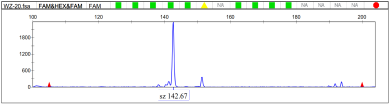 |
| --- | --- | --- |
