# Supplementary material for: Genetic diversity analysis of the invasive gall pest Leptocybe invasa (Hymenoptera: Apodemidae) from China
Source: PLoS One. 2021 Oct 14;16(10):e0258610. doi: 10.1371/journal.pone.0258610 (PMC8516283; doi:10.1371/journal.pone.0258610)
Supplement: S1 Table — (DOCX) [file pone.0258610.s002.docx]

**S1 Table. Samples GeneBank accession numbers used to construct the bayesian phylogenetic tree and haplotype network diagram**

| **Nation/Region** | **Haplotypes** | **GeneBank accession numbers** | **Sample size** |
| --- | --- | --- | --- |
| **Lineage A Total** | **Hap1-2** | **—** | **415** |
| Kenya | Hap1 | MH093190-MH093193、MH093226-MH093228、MH093410-MH093426  MH093440-MH093445、MH093446-MH093451、MH093427-MH093439 | 49 |
| Mozambique | Hap1 | MH093197-MH093199、MH093234-MH093238、MH093305-MH093309  MH093453-MH093475 | 36 |
| South Africa | Hap1 | MH093310-MH093324、MH093186、MH093241-MH093243、  MH093325-MH093339、 | 34 |
| Tunisia | Hap1 | KP233982-KP233984 | 3 |
| Uganda | Hap1 | MH093194-MH093196、MH093239-MH093240、MH093246  MH093252-MH093259、MH093394-MH093402 | 23 |
| Zimbabwe | Hap1 | MH093476 | 1 |
| Israel | Hap1 | MH093187-MH093189、MH093207-MH093210、MH093218-MH093223  MH093260-MH093279 | 33 |
| Laos | Hap1 | MH093200-MH093204、MH093280-MH093289、MH093452  MH093355、MH093349-MH093354、MH093345-MH093348  MH093340-MH093343 | 31 |
| Vietnam | Hap1 | MH093344 | 1 |
| Thailand | Hap1 | MH093211-MH093212、MH093217、MH093245、MH093247-MH093251  MH093372-MH093393 | 31 |
| Italy | Hap1 | MH093205-MH093206、MH093224-MH093225、MH093290-MH093304  MH093361-MH093371、KP233972-KP233978、KP233989 | 38 |
| Turkey | Hap1 | KP233954 | 1 |
| Brazil | Hap1 | MH093213-MH093216、MH093229-MH093233、MH093244  MH093403-MH093409、MH093356-MH093360、MH093477-MH093481 | 27 |
| Argentina | Hap1 | KP233979-KP233981 | 3 |
| China | Hap1 | MZ378903-MZ378924、MZ378926-MZ378932、MZ378934、MZ378963、MZ378987、MZ379017、MZ379019、MZ379024-MZ379025、MZ379027-MZ379029、MZ379031-MZ379033、MZ379038、MZ379049、MZ379057-MZ379059、MZ379061-MZ379064、MZ379067-MZ379072、MZ379074-MZ379076、MZ379079-MZ379088、MZ379120、MZ379122-MZ379136、MZ379138-MZ379144、MZ379146-MZ379151 | 99 |
| China | Hap2 | MZ379056、MZ379060、MZ379065、MZ379077-MZ379078 | 5 |
| **Lineage B Total** | **Hap3-25** | **—** | **411** |
| China | Hap3 | MZ378835-MZ378902、MZ378925、MZ378933、MZ378935-MZ378962、MZ378964-MZ378986、MZ378988-MZ379016、MZ379018、MZ379020-MZ379023、MZ379026、MZ379030、MZ379034-MZ379037、MZ379039-MZ379048、MZ379050-MZ379055、MZ379066、MZ379073、MZ379089-MZ379119、MZ379121、MZ379137、MZ379145、MZ379152-MZ379154、KP233985-KP233988、KP233990-KP233993、JQ289999-JQ280005 | 231 |
| Ghana | Hap3 | MH093117-MH093119 MH093168-MH093172 | 8 |
| South Africa | Hap3 | MH093111-MH093112、MH093180-MH093181 | 4 |
| Laos | Hap3 | MH093146-MH093147、MH093149-MH093152、MH093148 | 7 |
| Malaysia | Hap3 | MH093113-MH093116、MH093175-MH093179 | 9 |
| Vietnam | Hap3 | MH093139-MH093145、MH093173-MH093174 | 9 |
| Thailand | Hap3 | MH093120-MH093138、MH093153-MH093167 | 34 |
| Sunshine University | Hap4 | MH093006-MH093009 | 4 |
| Gardens_Point&  Miva&Noosa | Hap5 | MH093096-MH093110、MH093092-MH093093、MH093094-MH093095 | 19 |
| Hervey_Bay | Hap6 | MH093048-MH093051 | 4 |
| Hervey_Bay | Hap7 | MH093052 | 1 |
| Ingham | Hap8 | MH093080-MH093081 | 2 |
| Kenmore | Hap9 | MH093010-MH093042 | 33 |
| Maleny | Hap10 | MH093087-MH093088 | 2 |
| Marcella_Creek | Hap11 | MH093057-MH093062 | 6 |
| Mareeba,  North_Queensland | Hap12 | MH093063 | 1 |
| Miva | Hap13 | MH093079 | 1 |
| Nanango | Hap14 | MH093071-MH093074 | 4 |
| Takura | Hap15 | MH093075-MH093078 | 4 |
| Miva | Hap16 | MH093089-MH093091 | 3 |
| Nanango | Hap17 | MH093053 | 1 |
| Nanango | Hap18 | MH093064 | 1 |
| Nanango | Hap19 | MH093065-MH093070 | 6 |
| Takura&Walligan | Hap20 | MH093056、MH093054-MH093055 | 3 |
| Toolara | Hap21 | MH093043-MH093046 | 4 |
| Toolara | Hap22 | MH093047 | 1 |
| Tumoulin | Hap23 | MH093082-MH093086 | 5 |
| Walligan | Hap24 | MH093182-MH093184 | 3 |
| Walligan | Hap25 | MH093185 | 1 |
| **Lineage C Total** | **Hap26-27** | **—** | **5** |
| Maroochydore | Hap26 | MH093001 | 1 |
| Maroochydore | Hap27 | MH093002-MH093005 | 4 |
