# Supplementary material for: Genetic diversity analysis of the invasive gall pest Leptocybe invasa (Hymenoptera: Apodemidae) from China
Source: PLoS One. 2021 Oct 14;16(10):e0258610. doi: 10.1371/journal.pone.0258610 (PMC8516283; doi:10.1371/journal.pone.0258610)
Supplement: S2 Table — (DOCX) [file pone.0258610.s003.docx]

**S2 Table. Calibration results of linkage unbalance detection of 10 SSR primers of *L. invasa***

| **SSR association locus** | **P-Value** |
| --- | --- |
| c120771 & LiSS13 | 0.02025 |
| c120771 & c120888 | 0.02433 |
| c120771 & c124062 | 0.03062 |
| c120771 & c121749 | 0.06745 |
| c121749 & c127471 | 0.16154 |
| c124062 & c120888 | 0.30276 |
| c121460 & c127471 | 0.36251 |
| c124062 & c121749 | 0.42461 |
| c124062 & c127471 | 0.68036 |
| c69914 & c121749 | 0.70420 |
| c120888 & LiSS13 | 1.00000 |
| c120771 & c127471 | 1.00000 |
| c121749 & LiSS13 | 1.00000 |
| c124062 & LiSS5 | 1.00000 |
| c121749 & LiSS5 | 1.00000 |
| c120888 & LiSS5 | 1.00000 |
| c120771 & c123946 | 1.00000 |
| c121460 & LiSS5 | 1.00000 |
| c127471 & LiSS5 | 1.00000 |
| c120888 & c123946 | 1.00000 |
| c121460 & c121749 | 1.00000 |
| c120771 & c121460 | 1.00000 |
| c124062 & c121460 | 1.00000 |
| LiSS5 & LiSS13 | 1.00000 |
| c121749 & c123946 | 1.00000 |
| c127471 & LiSS13 | 1.00000 |
| c120771 & LiSS5 | 1.00000 |
| c69914 & LiSS13 | 1.00000 |
| c69914 & c127471 | 1.00000 |
| c69914 & LiSS5 | 1.00000 |
| c124062 & LiSS13 | 1.00000 |
| c127471 & c123946 | 1.00000 |
| c120888 & c121460 | 1.00000 |
| c123946 & LiSS5 | 1.00000 |
| c121460 & c69914 | 1.00000 |
| c124062 & c69914 | 1.00000 |
| c124062 & c123946 | 1.00000 |
| c120888 & c121749 | 1.00000 |
| c120771 & c69914 | 1.00000 |
| c123946 & LiSS13 | 1.00000 |
| c120888 & c69914 | 1.00000 |
| c69914 & c123946 | 1.00000 |
| c121460 & LiSS13 | 1.00000 |
| c120888 & c127471 | 1.00000 |
| c121460 & c123946 | 1.00000 |
