# Supplementary material for: Genetic diversity analysis of the invasive gall pest Leptocybe invasa (Hymenoptera: Apodemidae) from China
Source: PLoS One. 2021 Oct 14;16(10):e0258610. doi: 10.1371/journal.pone.0258610 (PMC8516283; doi:10.1371/journal.pone.0258610)
Supplement: S3 Table — (DOCX) [file pone.0258610.s004.docx]

**S3 Table. Hardy weinberg equilibrium test for 14 geographic populations of *L. invasa***

| Locus  POP | c120771 | c124062 | c120888 | c121460 | c69914 | c121749 | c127471 | c123946 | LiSS5 | LiSS13 |
| --- | --- | --- | --- | --- | --- | --- | --- | --- | --- | --- |
| SCDY | — | — | — | — | 0.006 | — | — | 0.000 | — | — |
| JXGZ | 0.00 | 0.263 | 0.059 | 0.928 | 0.000 | 0.034 | 0.558 | 0.000 | 0.036 | 0.000 |
| GXFCG1 | 0.000 | 0.000 | — | 0.000 | 0.003 | 0.000 | 0.000 | 0.000 | 0.219 | — |
| GXFCG2 | 0.000 | 0.000 | — | — | 0.310 | 0.000 | 0.000 | 0.000 | 0.927 | — |
| GXNN1 | 0.855 | — | 0.928 | — | 0.395 | — | — | 0.000 | — | — |
| FJSM | — | — | — | — | 0.240 | — | — | 0.000 | — | — |
| GXNN2 | 0.000 | 0.000 | 0.000 | 0.455 | 0.002 | — | 0.001 | 0.000 | — | — |
| SCPZH | 0.000 | 0.008 | 0.591 | — | 0.007 | 0.001 | 0.013 | 0.000 | 0.897 | 0.619 |
| GXWZ | 0.656 | 0.002 | 0.551 | 0.020 | 0.000 | 0.000 | 0.000 | 0.000 | 0.002 | 0.012 |
| GXLB | — | — | — | 0.897 | 0.001 | 0.000 | — | 0.000 | — | — |
| GXQZ | — | — | — | 0.824 | 0.083 | 0.014 | — | 0.014 | — | — |
| HNDZ | — | 0.046 | — | — | 0.261 | — | — | 0.230 | — | — |
| GXYL | — | 0.386 | — | — | 0.729 | — | — | 0.083 | — | — |
| YNKM | — | 0.564 | — | — | 0.861 | — | — | 0.386 | — | — |
| ALL POP. | High. sign. | High. sign. | 0.130 | 0.775 | High. sign. | High. sign. | High. sign. | High. sign. | High. sign. | 0.147 |

Note: POP: Populations; High. Sign.: high significant site; —: monomorphism site.
