# Supplementary material for: Genetic diversity analysis of the invasive gall pest Leptocybe invasa (Hymenoptera: Apodemidae) from China
Source: PLoS One. 2021 Oct 14;16(10):e0258610. doi: 10.1371/journal.pone.0258610 (PMC8516283; doi:10.1371/journal.pone.0258610)
Supplement: Attachment — Submitted filename: Manuscript-5.pdf [file pone.0258610.s005.pdf]

Genetic diversity analysis of invasive gall-pest *Leptocybe invasa* (Hymenoptera: Apodemidae) from China

Xin Peng[1], HanTang Wang[1], ChunHui Guo[1], Ping Hu[1], Lei Xu[1], Jing Zhou[1], ZhiRou Ding[1], ZhenDe Yang[1,2*]

1 Guangxi University, Forestry College, Nanning 530004, Peoples R China
2 Guangxi University, Forestry College, Guangxi Key Lab Forest Ecol & Conservat, Nanning 530004, Peoples R China

* Corresponding author：Zhende Yang, E-mail: dzyang68@126.com

Abstract

Leptocybe invasa Fisher et LaSalle is a global invasive pest that seriously damages *Eucalyptus* plants. To study the genetic diversity, genetic structure and introgression hybridization of *L. invasa* in China is of great significance for clarifying the breeding strategy, the future invasion and diffusion trend of *L. invasa* in China and developing scientific prevention and control measures. Genetic diversity and phylogenetic analysis of 320 *L. invasa* female adults from 14 geographic populations in China were conducted using 10 polymorphic microsatellite loci (SSR) and mitochondrial DNA cytochrome oxidase I gene sequences (COI). The results indicate that: (1) Bayesian phylogenetic tree and haplotype network diagram showed that only haplotype Hap3 existed in *L. invasa* lineage B in China, while haplotype Hap1 and Hap2 existed in lineage A, among which haplotype Hap2 was found for the first time. The nucleotide diversity and haplotype diversity of lineage A were higher than those of lineage B. (2)The SSR genetic diversity of Wuzhou Guangxi, Ganzhou Jiangxi and Panzhihua Sichuan populations were higher than that of the other 11 populations, and the SSR genetic diversity of lineage A was higher than that of lineage B. (3)The AMOVA analysis of mitochondrial COI data showed that 75.55% of the variation was among populations, and 99.86% of the variation was between lineage, while the AMOVA analysis of nuclear SSR data showed that 35.26% of the variation was among populations, and 47.04% of the variation was between lineage. There were obvious differences in the sources of variation between COI and SSR data. (4) The optimal K value of COI and SSR data in Structure analysis is 2, and PCoA analysis also divides the data set into two obvious categories. The UPMGA phylogenetic tree based on SSR data clustered 14 geographic species into two groups. The results of genetic structure analysis supported the existence of two lineage A and B in China. (5) The Structure analysis showed that there were obvious introgressive hybridization in Wuzhou Guangxi, Ganzhou Jiangxi, Panzhihua Sichuan and other populations. These results suggest that lineage introgressive hybridization have occurred in *L. invasa* population in China. The introgressive hybridization degree and genetic diversity of lineage A are obviously higher than that of lineage B. Lineage introgressive hybridization may be the driving force for further *L. invasa* invasion and diffusion in China in the future.

Deleted:
Deleted: China, and
Deleted: polymorphism
Deleted: obviously
Deleted: obviously
Deleted: 4)The
Deleted: 5)The
Deleted: introgression
Deleted: introgression
Deleted: introgression

Introduction

With the development of international trade and economic globalization, biological invasion has become a serious concern of the international community. China is one of the countries most

affected by invasive organisms in the world. At present, there are more than 640 kinds of invasive organisms in agriculture and forestry, including 130 kinds of invasive insects, which cause direct economic losses of more than 50 billion yuan every year [1].

Leptocybe invasa Fisher et LaSalle (Hymenoptera: Apodemidae) As an invasive gall-pest that damages more than 30 species of *Eucalyptus*, it mainly harms the branches and leaves of eucalyptus, causing typical lumplike galls on the veins, petioles and shoots of new leaves of eucalyptus. Serious infection can lead to plant death and huge economic losses. *L. invasa* was first discovered in the Middle East and Mediterranean in 2000 [2]. In 2014, it spread to 29 countries on the five continents [3]. In 2018, the number increased dramatically to 45 countries [4]. *L. invasa* was first discovered in China in 2007 in Dongxing, Fangchenggang, Guangxi, which is near the border with Vietnam [5]. Now it has spread to Guangxi, Hainan, Guangdong, Fujian, Sichuan, Jiangxi, Yunnan, Taiwan and other 8 provinces [3,6-8]. *L. invasa* has become an important invasive forest pest in eucalyptus worldwide with unprecedented scale and speed of invasion and spread.

L. invasa has three lineage A, B and C. lineage A is mainly distributed in Europe, the Middle East, South America and most parts of Africa. lineage A and B are distributed together in Laos, Thailand, Vietnam, South Africa and other places, while lineage C only appears in Australia [9-10]. Nugnes et al. first reported that *L. invasa* that invaded China belongs to lineage B [11]. However, Peng Xin et al. identified *L. invasa* from 14 geographical populations in China and found that there are both lineage A and lineage B in China, with a ratio of about 1:2 [12].

The main reproductive method of *L. invasa* is parthenogenesis, in which the genes of the female offspring are the same as their mother [2]. Some of the symbiotic bacteria in *L. invasa*, such as *Rickettsia*, can induce parthenogenesis [11]. Due to its superior parthenogenesis, *L. invasa* can rapidly expand its population in the invaded areas. In recent years, *L. invasa* males have been reported in Turkey (male/female ratio: 1:124) [13], India [14], China (male/female ratio: 1:126& 1:125) [15], Taiwan [16], Thailand [17] and other places successively. Liang Yiping's study found that the proportion of male *L. invasa* in Guangxi, China was 18% - 48%, which was significantly higher than the proportion reported in other reports [18]. Yang Xianxian studied the reproductive biology of *L. invasa* and found that there is mating behavior between male and female *L. invasa* in China, and offspring can be produced through bisexual reproduction [19]. Through the analysis of genetic diversity and genetic structure, Dittrich-Schröder et al. found that *L. invasa* in the Vietnamese population had introgressive hybridization between lineages [9].

Introgression is a phenomenon in which genetic material from one species is transferred to another species through hybridization and repeated backcrossing, also called introgressive hybridization [20]. Hybridization occurs in about 10 percent of all animals and 25 percent of all plants in nature [21], Hybridization often leads to introgression [22]. Hybridization is considered to be an important driving force for adaptive evolution of species [23-24], Introgression caused by hybridization has been reported in many animals, such as insects [25], mollusks [26], mammals [27], reptiles [28], amphibians [29], fish [30], etc. In recent decades, with the frequent occurrence of invasive species introduction events, the hybridization opportunities between invasive species and their relatives have increased, and the introgressive hybridization progeny may be more invasive, leading to the extinction of the local parent groups [31-32]. Introgressive hybridization is easy to occur between lineage due to their close genetic relationships. Introgression leads to the rapid improvement of genetic diversity, which increases the fitness of lineage in new environments, and promotes the spread of lineage and population expansion [30,33-34]. The mixing of *L. invasa* lineage may lead to introgression between lineage and the emergence of new genotypes that are more suitable for the environment [9]. In this paper, SSR and COI molecular markers were used to analyze the genetic diversity, genetic structure and introgressive

Deleted: seriously

Deleted: introgression

Deleted: Introgression

hybridization of *L. invasa* in China, aiming to provide a basis for the prediction and scientific prevention and control of *L. invasa* invasion and spread trend in China.

Deleted: introgression

Methods

Sample collection and DNA extraction

In this study, a total of 320 female adult *L. invasa* samples from 14 geographic populations in 6 provinces of China were collected during 2016-2020 (Table 1). All the samples were collected from DH201-2 (*Eucalyptus grandis* × *Eucalyptus tereticornis*) (Myrtales: Myrtaceae), except for GXNN2 (*Eucalyptus exserta*) (Myrtales: Myrtaceae).

Commented [MOU1]: How many samples were obtained per location. This should be stated here or in the table

Fresh shoots of eucalyptus with gall were collected from each geographic population, the branches were placed in a plastic bottle filled with water to retain freshness and transferred into a sealed net cage (40 cm×40 cm×80 cm) at room temperature to keep the adults from escaping. The water in the plastic bottle was renewed daily until the emergence of *L. invasa* adults. Sexes were identified by morphological observation [3]. Samples of female adults from each geographic population were placed into tubes with anhydrous ethanol, labeled and stored in a refrigerator at -80℃. In order to avoid contamination, only single geographical population *L. invasa* samples were cultivated each time, and the remaining branches were treated in time after the culture. Thirty-two samples were randomly selected from the samples collected from each geographic population for subsequent molecular analysis, and Some populations with insufficient samples use the entire samples.

Deleted: The

The animal tissue DNA extraction kit (Beijing Tsingke Biological Technology) was used to extract the total genomic DNA of single *L. invasa* female adults according to the instructions. Nanodrop spectrophotometer was used to measure the quality and concentration of DNA at 260 nm/280 nm and 260 nm/230 nm ratios, and the qualified DNA was stored in a refrigerator at -20℃ for later use.

Commented [MOU2]: You are writing for an audience some may not know what nanodrop is.

Mitochondrial COI gene sequence download

320 COI sequences of 14 geographical populations in China (MZ378835 - MZ379154) [12] and 511 COI sequences of 18 countries in 5 continents (MH093001-MH093481, JQ289999-JQ290005, KP233972-KP233993, KP233954) [9,11] were downloaded from GenBank molecular database.

Microsatellite amplification and capillary electrophoresis genotyping

Reference was made to the polymorphic SSR primers of *L. invasa* developed by Dittrich-Schröder et al. [9] and Peng Xin et al. [12] (Table 2). The 5' end of the Forward primer was labeled with four kinds of fluorescence (FAM, ROX, HEX and TAMAR) (Beijing Tsingke Biological Technology). The sample DNA used was consistent with mitochondrial sequencing. The PCR reaction system was 25μL, including 2×T5 Super PCR Mix 12.5μL (Beijing Tsingke Biological Technology), forward fluorescent primer and reverse primer (diluted to 10 μm) 1.0μL each, DNA template 1.0μL, ddH_2O 9.5μL. The PCR reaction procedure was pre-denaturation at 98℃ for 3min, denaturation at 98℃ for 10s, annealing at suitable temperature for 10s, extension at 72℃ for 10s, 35 cycles and extension at 72℃ for 2min. PCR products were sent to Beijing Tsingke Biological Technology for capillary electrophoresis genotyping using QIAXCEL nucleic acid analysis system (QIAGEN, Irvine, USA) (S1 Fig).

Deleted:

Bayesian phylogenetic tree and haplotype network diagram

construction

831 COI sequence data downloaded from GenBank molecular database were input into Notepad++ v6.5 software and saved in FASTA format（S1 Table）. The COI sequences were Mafft alignment using PhyloSuite v1.1.1 [36] and conserved sequences were extracted using Gblocks. The optimal model of Bayesian tree was found through the Model Finder program in PhyloSuite v1.1.1 software, and the optimal model was used to construct the Bayesian phylogenetic tree. The generation number was set as 100 million, and 3 replicates were run. *L. invasa* COI gene sequence haplotype network was constructed using Popart v1.7 software（http://popart.otago.ac.nz）.

Commented [MOU3]: This statement needs to be revised

Genetic diversity analysis

Genetic diversity analysis based on SSR data: The peak data of capillary electrophoresis was input into Excel in GeneAlex v6.5 [37] format, and the format conversion required for subsequent software was completed using GeneAlex v6.5 [37] plug-in. Genetic diversity analysis software was used to analyze the genetic diversity of (1) different geographic populations (N =320), (2) lineage A (N = 104) (3) lineage B (N = 216). Arlequin v3.5 [38] was used to calculate genetic diversity indexes, including total number of alleles, average number of alleles, number of polymorphic loci, average observed heterozygosity, average expected heterozygosity and average genetic diversity. Arlequin v3.5 [38] was used to perform molecular analysis of variance (AMOVA) to quantitatively analyze the genetic variation among populations, within populations, between lineage and within lineage. Genepop v4.2 [39] was used to detect Linkage disequilibrium, And a significant correction of Linkage disequilibrium through the "sequential Bonferroni correction" [40] (S2 Table). Genepop v4.2 [39] was used to detect Hardy-Weinberg equilibrium at each site (S3 Table), and Cervus v3.0 [41] software was used to calculate polyphic information content (PIC) at each locus.

Commented [MOU4]: This statement is confusing please re-write

Commented [MOU5]: Do you mean Polymorphic?

Deleted: i

Genetic diversity analysis based on COI data: The haplotype diversity and nucleotide diversity of different lineage and all samples were calculated using DNAsp v6 [42]. Arlequin v3.5 [38] was used to analyze the molecular variance of *L. invasa* within and among populations, within and between lineage in China.

Genetic structure analysis

Population v1.2.32 software (http://bioinformatics.org/project/?group_id=84) was used to construct UPMGA population evolutionary tree based on SSR data. Structure version 2.3 (http://pritch.bsd.uchicago.edu/structure.html), which applies a Bayesian clustering method, was used to indicate the number of populations or clusters (K) based on genetic identity of the individuals and without geographic prior. An admixture model with correlated allele frequencies was implemented because it was more likely to detect subtle population structure. Tests included 20 repetitions for a range of K-values from 1 to 10. For the Markov chain Monte Carlo (MCMC) procedure, the ‘burn in’ period was set to 100000 iterations followed by 100000 samples. Structure Harvester [43] is used to determine the likelihood value and ΔK [44] changes to verify the optimal K value, which represents the most likely clustering of individuals. Visualize the results through the website (http://pophelper.com/). Principal co-ordinate analysis (PCoA) was performed on SSR and COI data using GenAlEx v6.5 [37].

Results

Bayesian phylogenetic tree and haplotype network diagram

Bayesian phylogenetic tree and haplotype network were constructed by using 831 605bp COI gene sequences downloaded from GenBank. The Bayesian tree and haplotype network diagram shows that there are three lineage of *L. invasa*: A(Hap1-Hap2), B(Hap3-Hap25), and C(Hap26-Hap27). Haplotype Hap4-Hap25 and Hap26-Hap27 were found only in Australia, while haplotype Hap3 and Hap1 was widely distributed in countries other than Australia where where *L. invasa* is invaded except Australia. Two lineage, lineage A and lineage B, exist in China, including Hap1, Hap2 and Hap3, among which Hap2 was first found in GXFCG1 population in China (Fig 1-2).

Deleted: genebank

Genetic diversity

The genetic diversity analysis of COI data showed (Table 3) that the base diversity and nucleotide diversity of *L. invasa* lineage A were significantly lower than lineage B and lineage C. There was no base mutation in the COI gene sequence of lineage B in China, and lineage A only had one base mutation site. The nucleotide diversity and haplotype diversity at the lineage level are both at a low level, indicating that *L. invasa* in China has experienced population expansion recently. AMOVA analysis (Table 4) showed that the genetic variation among and within populations accounted for 75.55% and 24.45%, respectively. The genetic variation between and within lineage accounted for 99.86% and 0.14%, respectively. Genetic variation is concentrated among populations and lineage.

Diversity analysis of SSR data shows (Table 5), At the population level, JXGZ had the most alleles, GXWZ had the highest observed heterozygosity and expected heterozygosity, and the genetic diversity of GXWZ, JXGZ and SCPZH populations was significantly higher than that of the other 11 populations. At the level of lineage, the total number of alleles of lineage A and B was similar, but the observed heterozygosity, expected heterozygosity, number of polymorphic sites and average genetic diversity of lineage A were significantly higher than those of lineage B. AMOVA analysis (Table 4) showed that the genetic variation among and within populations accounted for 38.61% and 61.39%, respectively. The genetic variation between and within lineage accounted for 48.86% and 51.14%, respectively. Compared with the COI gene sequence, the genetic variation of SSR was concentrated within the populations and lineage.

Genetic structure

In the UPMGA population evolutionary tree constructed based on SSR data (Fig 3), GXFCG1, JXGZ, GXWZ and SCPZH populations were clustered into one branch, and the other populations were clustered into another branch, which was consistent with the results of Structure analysis.

The principal co-ordinates analysis (PCoA) of COI data showed that lineage A and lineage B were clearly separated, and co-ordinates 1 and 2 represented 99.65% and 0.35% variation percentages, respectively (Fig 4). The principal co-ordinate analysis of SSR data showed that there was a mixing region between lineage A and B, and coordinates 1 and 2 represented 44.32% and 12.26% variation percentages, respectively (Fig 4), indicating the presence of gene introgression between lineages.

Deleted:

Deleted:

Deleted: lineage

The optimal K value of structure analysis based on COI data is 2, The black and yellow represent the samples of lineage A and lineage B, respectively. The lineage A and B appeared simultaneously in 7 populations, namely GXFCG1, JXGZ, GXWZ, SCPZH, GXFCG2, GXNN1 and GXNN2 (Fig 5).

The optimal K value of structure analysis based on SSR data is also 2. Black and yellow

represent the genes of lineage A and lineage B, respectively. Lineage mixing appears in part of the samples, indicating that the samples are introgression hybrids. The samples of GXFCG1, JXGZ, GXWZ and SCPZH populations were dominated by the genes of lineage A, while the other populations were dominated by the genes of lineage B. Different degrees of introgressive hybridization appeared in JXGZ, GXWZ, SCPZH, GXNN2, GXQZ, GXYL, GXLB and HNDZ populations. *L. invasa* in GXWZ and SCPZH populations were almost replaced by their introgressed hybrids (Fig 5).

Discussion

We captured *L. invasa* female adult samples from 14 geographic populations in 6 provinces of China, covering most of the *L. invasa* hazard areas in China. In particular, Guangxi Province, where the *L. invasa* outbreak first occurred, has set up multiple sampling sites to speculate on its possible transmission route. Because all susceptible varieties of eucalyptus have been replaced with highly resistant varieties in Guangdong Province, we did not collect the samples of *L. invasa* from Guangdong population, which indicated that the comprehensive promotion of resistant varieties has a very good effect on the control of *L. invasa*. There are two lineage of *L. invasa* in China: lineage A and lineage B [12], which are consistent with the lineage in other countries where *L. invasa* is invaded in the world. However, a new haplotype HAP2 was found in the lineage A of the Chinese population by Bayesian clustering analysis, which is of guiding significance for the traceability of lineage A. The lineage structure of *L. invasa* in China is similar to that in Southeast Asia, which indicates that *L. invasa* in China probably originated from Southeast Asia.

When selecting appropriate pest control methods, it is necessary to consider the genetic diversity, genetic structure and gene flow of the pest population, which can help us predict the future distribution of invasive pests, the possibility of hybridization between different lineage, and the ability to overcome the resistance of different host plants. Mixtures between closely related species are of particular concern, as introgression within a mixed population can rapidly increase genetic diversity within the population, thus enabling pests to invade previously unsuitable environments or to overcome control measures [45-46]. The progeny of parthenogenesis is genetically the same as the mother, and the genetic diversity of its population is mainly determined by a few founders. The genotypes of its progeny are highly consistent, which is the root cause of the low polymorphism of *L. invasa* SSR loci [9]. The genetic structure analysis of *L. invasa* of different geographical populations in China using Structure and PCoA showed that there was obvious introgressive hybridization in China, which was similar to the genetic Structure analysis results of Dittrich-Schröder et al. in Laos [9]. Introgressive hybridization among lineage significantly increased genetic diversity in some geographic populations, such as Wuzhou in Guangxi, Panzhihua in Sichuan and Ganzhou in Jiangxi. In AMOVA analysis, the variation percentage within lineage based on COI data (99.84%) was significantly higher than that based on SSR data (44.86%). This phenomenon of inconsistency in the source of nucleoplasmic gene variation also proved that introgressive hybridization occurred between lineages. According to the genetic structure of the species, there were heterozygous samples in both lineage A and B, which indicated that the introgression between lineage of *L. invasa* may be bidirectional. Le et al. speculated that *L. invasa* lineage B might be more inclined to produce males [4]. Therefore, in the process of introgressive hybridization, lineage B is more likely to infiltrate genes into lineage A through sexual reproduction, which makes the genetic diversity and introgressive hybridization degree of lineage A significantly higher than that of lineage B. In addition, survival pressure may also be an important factor affecting *L. invasa* reproductive strategy. When food supply is

Deleted: ,
Deleted: introgsive
Deleted: introgressional
Deleted: introgression
Deleted: e
Deleted: I
Deleted: ntrogated
Deleted: introgsive
Deleted: lineage
Deleted: introgression
Deleted: introgression

insufficient, *L. invasa* will increase male differentiation, thus reducing reproductive ability and the number of offspring. For example, the proportion of male collected from *E. exserta* was 1.7-1.8 times that of DH201-2 (*E. grandis* ×*E. tereticornis*) [18]. In this study, the introgressive hybridization degree of Guangxi Nanning 2 population was significantly higher than that of Guangxi Nanning 1 population, which also indicated that the host plants would affect the reproduction strategy of *L. invasa*. In recent years, China has vigorously promoted resistant varieties of eucalyptus, resulting in the lack of suitable hosts for *L. invasa* and insufficient food supply, leading to the transformation of *L. invasa* reproduction strategy and more frequent sexual reproduction in the population.

Deleted: introgsive

The emergence of introgressive hybridization in lineage has sounded the alarm for the control of *L. invasa* in China. Hybrids have been widely distributed in several populations in China. Among them, *L. invasa* in Guangxi Wuzhou population and Sichuan Panzhihua population were almost replaced by their introgressed hybrid offspring, which indicates that there is obvious heterosis in *L. invasa* population. For most organisms, heterosis can only be maintained for one generation and is difficult to be inherited steadily. However, *L. invasa* can rapidly expand the number of offspring of dominant hybrids through parthenogenesis, thus breaking the reproductive barrier to avoid inbreeding decline and maintaining heterosis in the population for a long time. Hybrids can form large populations independent of their parents in a short period of time. Once a hybrid is able to adapt to the new environment, It can invalidate the previously dependent resistant varieties and specific natural enemies, And in a short time again outbreaks of insect pests. There are a large number of clonal eucalyptus forests in China, which will become a huge hidden trouble for *L. invasa* control. Especially the remaining stands of susceptible eucalyptus species such as DH201-1, DH201-2 (*E. grandis* × *E. tereticornis*) and *E. exserta* margin. *L. invasa* can quickly establish a large population on susceptible host plants. The aggregation of a large number of progenies increase the chance of gene exchange between lineage, and susceptible host plants accelerate the generation replacement and evolution rate of pests. Eucalyptus susceptible species can not only serve as the source of *L. invasa*'s continuous spread, but also become a hotbed for *L. invasa* to adapt to the environment through genetic variation. With the intensification of *L. invasa* lineage mixing, gene penetration between lineage will become a trend in the future. *L. invasa* flexibly applies two reproductive strategies to adapt to the environment, which will increase the difficulty of its prevention and control.

Deleted: introgression

Deleted: ion

Deleted: progeny

Deleted: increases

Conclusion

Introgressive hybridization between *L. invasa* lineage A and B has occurred in many geographical populations in China. The genetic diversity and introgression degree of *L. invasa* lineage A were significantly higher than that of lineage B. lineage introgressive hybridization may be the driving force for *L. invasa* to invade and spread in China in the future.

Deleted: Introgression

Deleted: introgression

Supporting information

S1 Fig. *L. invasa* SSR capillary electrophoresis peak.
(DOCX)

S1 Table. *L. invasa* Samples GenBank accession numbers used to construct the Bayesian phylogenetic tree and haplotype network diagram.
(DOCX)

Deleted: GeneBank

Deleted: bayesian

S2 Table. Calibration results of linkage unbalance detection of 10 SSR primers of *L. invasa.*
(DOCX)

S3 Table. Hardy Weinberg equilibrium test for 14 geographic populations of *L. invasa.*
(DOCX)

Deleted: weinberg

Acknowledgements

We thank Lei Xu and Hantang Wang for insect collection. And thank the subsidization of the National Natural Science Foundation of China (No.31971664, 31560212) and the Guangxi Natural Science Foundation (No.2018GXNSFAA294008, 2018GXNSFDA281004).

Author Contributions

Xin Peng conducted molecular genetic studies, performed sequence alignment, experiments and drafted the manuscript. Lei Xu, Hantang Wang and Ping Hu collected samples and participated in some experiments. Xin Peng participated in all experiments. Zhende Yang and Chunhui Guo designed and conceived of the study; Ping Hu also helped draft the manuscript. All authors read and approved the final manuscript.

Reference

1. Wan F H, Jiang M X, Zhan A. Biological Invasions and Its Management in China. Netherlands: Springer Nature. 2017. https://doi.org/10.1007/978-94-024-0948-2
2. Mendel Z, Protasov A, Fisher N, et al. Taxonomy and biology of *Leptocybe invasa* gen. & sp. n. (Hymenoptera: Eulophidae), an invasive gall inducer on Eucalyptus. Aust J Entomol. 2004; 43: 101-113. https://doi.org/10.1111/j.1440-6055.2003.00393.x
3. Zheng X L, Li J, Yang Z D, et al. A review of invasive biology, prevalence and management of *Leptocybe invasa* Fisher & La Salle (Hymenoptera: Eulophidae: Tetrastichinae). Afr Entomol. 2014; 22: 68-79. https://doi.org/10.4001/003.022.0133
4. Le N H, Nahrung H F, Griffiths M, et al. Invasive *Leptocybe spp*. and their natural enemies: Global movement of an insect fauna on eucalypts. Biol Control. 2018; 125: 7-14. https://doi.org/10.1016/j.biocontrol.2018.06.004
5. Wu Y J, Jiang X J, Li D W, et al. *Leptocybe invasa*, a New Invasive Forest Pest Making Galls on Twigs and Leaves of *Eucalyptus* Trees in China (Hymenoptera:Eulophidae) (in Chinese). Sci Silv Sin. 2009; 45: 161-163+182-183 .
6. Zheng X L, Huang Z Y, Li J, et al. Reproductive Biology of *Leptocybe invasa* Fisher & La Salle (Hymenoptera: Eulophidae). Neotrop Entomol. 2018; 47(1): 19-25. https://doi.org/10.1007/s13744-017-0502-6
7. Zheng X L, Lin K, Huang Z Y, et al. Offspring sex ratio and reproductive tactics of *Leptocybe invasa* (Hymenoptera: Eulophidae): testing the effect of environmental characteristics. Int J Trop Insect Sci. 2018; 38(4): 394-399. https://doi.org/ 10.1017/S1742758418000127
8. Zhang H F, Studies on Mechanism of *Leptocybe invasa* Infestation and Chemical Defense of *Eucalyptus* (in Chinese). Fuzhou: Fujian Agric For Univ. 2013.
9. Dittrich-Schröder G, Hoareau T B, Hurley B P, et al. Population genetic analyses of complex global insect invasions in managed landscapes: a *Leptocybe invasa* (Hymenoptera) case study. Biol Invasions. 2018; 20: 2395-2420. https://doi.org/10.1007/s10530-018-1709-0
10. Wondafrash M, Slippers B, Nambazimana A, et al. Distribution and genetic diversity of five invasive pests of Eucalyptus in sub-Saharan Africa. Biol Invasions. 2020; 22: 2205-2221.

https://doi.org/10.1007/s10530-020-02250-4
11. Nugnes F, Gebiola M, Monti M M, et al. Genetic Diversity of the Invasive Gall Wasp *Leptocybe invasa* (Hymenoptera: Eulophidae) and of its Rickettsia Endosymbiont, and Associated Sex-Ratio Differences. Plos One. 2015; 10: e0124660. https://doi.org/10.1371/journal.pone.0124660 PMID: 25970681
12. Peng X, Wang H T, Guo C H, et al. EST-SSR Development and cryptic species Identification for the invasive gall-pest *Leptocybe invasa* (Hymenopetra: Eulophidae) (in Chinese). Sci Silv Sin, 2021. in press .
13. Doğanlar O. Occurrence of *Leptocybe invasa* Fisher & La Salle, 2004 (Hymenoptera: Chalcidoidea) on Eucalyptus camaldulensis in Turkey, with a description of the male sex. Zool Middle East. 2005; 35: 112-114. https://doi.org/10.1080/09397140.2005.10638116
14. Gupta A, Poorani J. Taxonomic studies on a collection of Chalcidoidea (Hymenoptera) from India with new distribution records. J Threat Taxa. 2009; 1: 300-304. https://doi.org/10.11609/JoTT.o1861.300-4
15. Chen H Y, Yao J M, Xu Z F. First description of the male of *Leptocybe invasa* Fisher & LaSalle (Hymenoptera: Eulophidae) from China (in Chinese). J Environ Entomol. 2009; 31: 285-287 . https://10.3969 /j.issn.1674-0858.2009.03.015
16. Tung G S, La S J. Pest Alert-a newly discovered invasion of gall-forming wasps, *Leptocybe invasa*(Fisher & La Salle), on eucalyptus trees in Taiwan. Formos Entomol. 2010; 30: 241-245. https://doi.org/10.6661/TESFE.2010018
17. Sangtongpraow B, Charernsom K, Siripatanadilok S. Longevity, fecundity and development time of Eucalyptus gall wasp, *Leptocybe invasa* Fisher and La Salle (Hymenoptera: Eulophidae) in Kanchanaburi province, Thailand. Thai J Agric Sci. 2011; 44: 155-163.
18. Liang Y P, Li J Y, Wen X J, et al. Observation on the male ratio of *Leptocybe invasa* adult (in Chinese). For Pest Dis. 2010; 29: 21-22+18 .
19. Yang X X. Reproductive Biology of *Leptocybe invasa* Fisher & LaSalle (in Chinese). Wuhan: S China Agric Univ, 2017 .
20. Anderson E. Introgresive hybridization. Biol Rev. 1953; 28: 280-307.
21. Mallet J. Hybridization as an invasion of the genome. Trends Ecol & Evol. 2005; 20: 229-237. https://doi.org/10.1016/j.tree.2005.02.010
22. Bowley S R, Taylor N L. Introgressive hybridization. In: Christie ER, eds. CRC Handbook of Plant Science in Agriculture, vol. 1. Boca Raton: CRC Press, 1987: 23-59.
23. Kagawa K, Takimoto G. Hybridization can promote adaptive radiation by means of transgressive segregation. Ecol Lett. 2018; 21: 264-274. https://doi.org/10.1111/ele.12891
24. Seehausen O. Hybridization and adaptive radiation. Trends Ecol & Evol. 2004; 19: 198-207. https://doi.org/10.1016/j.tree.2004.01.003
25. Gao Shang, Jiang G F, Fan B B. Gene introgreesion between two species of sympatric bamboo locusts based on microsatellite markers (in Chinese). J Environ Entomol. 2020; 42: 566-572 . https://doi.org/10.3969 /j.issn.1674-0858.2020.03.7
26. Yang Q Q, He C, Liu G F, et al. Introgressive hybridization between two non-native apple snails in China: widespread hybridization and homogenization in egg morphology. Pest Manag Sci. 2020; 76: 4231-4239. https://doi.org/10.1002/ps.5980
27. Antoniou A, Frantzis A, Alexiadou P, et al. Evidence of introgressive hybridization between Stenella coeruleoalba and Delphinus delphis in the Greek Seas. Mol Phylogenet Evol. 2018; 129: 325-337. https://doi.org/10.1016/j.ympev.2018.09.007
28. Zarza E, Reynoso V H, Faria C M A, et al. Introgressive hybridization in a Spiny-Tailed Iguana, Ctenosaura pectinata, and its implications for taxonomy and conservation. PeerJ. 2019; 7: e6744. https://doi.org/10.7717/peerj.6744

29. Vallinoto M, Cunha D B, Bessa-Silva A, et al. Deep divergence and hybridization among sympatric Neotropical toads. Zool J Linn Soc. 2017; 180: 647-660. https://doi.org/10.1093/zoolinnean/zlw001
30. Zhu S Q, Cao X Y, Zhao J L, et al. Hybridization and introgression between *Siniperca chuatsi* and *S. kneri* from two lakes in middle Yangtze River (in Chinese). J Shanghai Ocean Univ. 2020; 29: 1-8 . https://doi.org/10. 12024 /jsou. 20190402614
31. Yamaguchi R, Yamanaka T, Liebhold A M, et al. Consequences of hybridization during invasion on establishment success. Theor Ecol. 2019; 12: 197-205. https://doi.org/10.1007/s12080-019-0415-6
32. Todesco M, Pascual M A, Owens G L, et al. Hybridization and extinction. Evol Appl. 2016; 9: 892-908. https://doi.org/10.1111/eva.12367
33. Chu D, Guo D, Pan H P, et al. Cryptic invasion of alien species: types and effects (in Chinese). Acta Entomol Sin. 2012; 55: 218-225. https://doi.org/10.16380/j.kcxb.2012.02.009
34. Queffelec J, Allison J D, Greeff J M, et al. Influence of reproductive biology on establishment capacity in introduced Hymenoptera species. Biol Invasions. 2021; 23: 387-406. https://doi.org/10.1007/s10530-020-02375-6
35. Folmer O, Black M, Hoeh W, et al. DNA primers for amplification of mitochondrial cytochrome c oxidase subunit I from diverse metazoan invertebrates. Mol Mar Biol Biotechnol. 1994; 3: 294-299. PMID: 7881515
36. Zhang D, Gao F L, Jakovlić I, et al. PhyloSuite: An integrated and scalable desktop platform for streamlined molecular sequence data management and evolutionary phylogenetics studies. Mol Ecol Resour. 2020; 20: 348-355. https://doi.org/10.1111/1755-0998.13096
37. Peakall R, Smouse P E. GenAlEx 6.5: genetic analysis in Excel. Population genetic software for teaching and research—an update. Bioinformatics. 2012; 28: 2537-2539. https://doi.org/10.1093/bioinformatics/bts460
38. Excoffier L, Lischer H E L. Arlequin suite ver 3.5: a new series of programs to perform population genetics analyses under Linux and Windows. Mol Ecol Resour. 2010; 10: 564-567. https://doi.org/10.1111/j.1755-0998.2010.02847.x
39. Raymond M, Rousset F. GENEPOP (Version 1.2): Population Genetics Software for Exact Tests and Ecumenicism. J Hered. 1995; 86: 248-249.
40. Rice W R. ANALYZING TABLES OF STATISTICAL TESTS. Evolution. 1989; 43: 223-225.
41. Kalinowski S T, Taper M L, Marshall T C. Revising how the computer program cervus accommodates genotyping error increases success in paternity assignment. Mol Ecol. 2007; 16: 1099-1106. https://doi.org/10.1111/j.1365-294X.2007.03089.x
42. Rozas J, Ferrer-Mata A, Sánchez-DelBarrio J C, et al. DnaSP 6: DNA Sequence Polymorphism Analysis of Large Data Sets. Mol Biol Evol. 2017; 34: 3299-3302. https://doi.org/10.1093/molbev/msx248
43. Earl D A, VonHoldt B M. STRUCTURE HARVESTER: a website and program for visualizing STRUCTURE output and implementing the Evanno method. Conserv Genet Resour. 2012; 4: 359-361. https://doi.org/10.1007/s12686-011-9548-7
44. Evanno G, Regnaut S, Goudet J. Detecting the number of clusters of individuals using the software structure: a simulation study. Mol Ecol. 2005; 14: 2611-2620. https://doi.org/10.1111/j.1365-294X.2005.02553.x
45. Dittrich-Schroeder G, Hurley B P, Wingfield M J, et al. Invasive gall-forming wasps that threaten non-native plantation-grown Eucalyptus: diversity and invasion patterns. Agric For Entomol. 2020; 22: 285-297. https://doi.org/10.1111/afe.12402

46. Garnas J R, Auger-Rozenberg M, Roques A, et al. Complex patterns of global spread in invasive insects: eco-evolutionary and management consequences. Biol Invasions. 2016; 18: 935-952. https://doi.org/10.1007/s10530-016-1082-9
